# Prevalence of hepatitis C virus in adult population in the Czech Republic – time for birth cohort screening

**DOI:** 10.1371/journal.pone.0175525

**Published:** 2017-04-13

**Authors:** Roman Chlibek, Jan Smetana, Renata Sosovickova, Peter Gal, Petr Dite, Vlasta Stepanova, Lenka Pliskova, Stanislav Plisek

**Affiliations:** 1 Department of Epidemiology, Faculty of Military Health Sciences, University of Defence, Hradec Kralove, Czech Republic; 2 Military Health Institute, Prague, Czech Republic; 3 Department of Clinical Microbiology, University Hospital Hradec Kralove, Czech Republic; 4 Department of Clinical Chemistry and Diagnostics, University Hospital Hradec Kralove, Czech Republic; 5 Department of Infectious Diseases, Charles University Medical Faculty and University Hospital Hradec Kralove, Czech Republic; University of North Carolina at Chapel Hill School of Dentistry, UNITED STATES

## Abstract

Chronic hepatitis C is curable disease. Low detection rate could be one of the reasons of poor treatment uptake. It is important to identify HCV prevalence and anti-hepatitis C virus (HCV) positive patients in population by effective screening strategy such as risk-based or birth cohort screening programs. There are no national population-based estimates of the HCV prevalence in the Czech Republic (CZ). The most recent seroprevalence survey determined a prevalence of positive anti-HCV antibodies of 0.2% (in 2001). The aim of the study was to determine the seroprevalence of HCV, HCV viraemia and HCV genotype in the CZ adult population. We also estimated the number of persons living with chronic hepatitis C in CZ. The examined group included 3000 adults, 18–90 years of age enrolled in 2015. All serum samples were examined to determined anti-HCV antibodies positivity, HCV-RNA positivity and genotypes. Of the 3000 samples, 50 were found to be anti-HCV-positive, for a seroprevalence of 1.67% (2.39% in males, 0.98% in females). The overall prevalence of positive HCV RNA was 0.93%: 1.5% in males, 0.39% in females. HCV genotype (GT) 1a was determined in 25%, GT 1b in 25% and GT 3a in 46%. Since 2001, the HCV seroprevalence has increased 8-fold. The highest HCV seroprevalence occurred in males aged 30–44 years. We can estimate that there are more than 140,000 people with HCV antibodies and more than 80,000 people with chronic hepatitis C living in the CZ. The introduction of birth cohort HCV screening could be beneficial for the country.

## Introduction

Hepatitis C virus (HCV) infection is a severe inflammatory necrotic liver disease that is frequently asymptomatic or with non-specific symptoms in its acute phase. It is the chronic form of HCV that causes significant morbidity and mortality with a risk of liver cirrhosis and subsequently hepatocellular cancer [1–4]. The disease is frequently unrecognized and undiagnosed in its acute phase, and the first clear symptoms may indicate serious disease. The worldwide prevalence of hepatitis C is 3%. It is estimated that there are 180–200 million people infected with HCV [5]. In Europe alone, there are 9 million people with chronic hepatitis C, and the prevalence ranges from 0.5% to 3.5%, with the highest prevalence rates in the Mediterranean region [6,7]. The distribution of HCV genotypes is variable. In Europe and in the United States of America, genotype 1 (GT1) is the most common; however, there is a growing significance of genotype 3 [8–16] in Europe, including in the Czech Republic (CZ). The prevalence of chronic hepatitis in the CZ is estimated to be low [17–19]. The most recent prevalence data in the CZ are from a seroprevalence survey conducted more than 15 years ago (in 2001), in which the prevalence was reported to be 0.2% [20]. No previous surveys have been conducted since then among adults in the CZ only, and we suspect a higher prevalence of HCV infection and an increase in subtype GT3 compared to the most recent data [21–23]. There is no screening of population groups in the CZ, which would be similar to “baby boomer” testing in the USA [24]. Only a small number of patients with hepatitis C are recognized in time and treated, and we expect that 20–50% of patients are not diagnosed or treated at all. One of the barriers to early diagnosis and treatment initiation is the low awareness and knowledge of physicians, along with insufficient screening. Undiagnosed hepatitis C infection frequently exists in the primary care setting [25]. With an ageing population, the significant impact of chronic HCV infection on the health care system is expected to increase in the Czech Republic as well [26]. Seroprevalence surveys of the general population are the gold standard for assessing the number of HCV infected within a country if there are no other surveillance databases available [27].

The main objective of the study was to determine the prevalence of specific anti-HCV antibodies and of positive HCV RNA in serum samples of the general adult population in the Czech Republic. The secondary objective was to determine the genotypes of HCV RNA-positive patients, to determine the prevalence of anti-HCV and HCV RNA in persons with high-risk behaviour and to determine the number of persons with a history of acute or chronic HCV infection.

## Materials and methods

### Study design and population

The study was performed as a prospective multicenter observation seroprevalence analytic study. There were three research centres that enrolled a total of 3000 healthy adult subjects ≥ 18 years of age. Subjects were enrolled into four age groups (18–29; 30–44; 45–59 and ≥ 60 years) to correspond with the age stratification and demographic proportion of these age groups in the general population. This study included a sample of the general adult population that agreed to participate in the clinical research study at the invitation of the research centres based on an advertising campaign in the media. The subject were enrolled on the “first-come, first served” principle. The enrolment period lasted from February 2015 to September 2015. All subjects had 5 ml of venous blood drawn. Each participant also completed a questionnaire concerning their risk factors and risk behaviour. These risk factors and behaviours included a history of blood transfusions before 1992, transplants, surgical intervention, infectious hepatitis C, injection drug use, sexual promiscuity, sexually transmitted diseases, tattoos, piercings or employment in healthcare.

The study was approved by a multicenter ethics committee and was conducted in full compliance with the principles of good clinical and laboratory practice, the conclusions of the international conference on harmonization and the Declaration of Helsinki. Each of the subjects provided written informed consent to participate in the study prior to enrolment.

### Testing anti-HCV antibodies

Serum samples were analysed by testing anti-HCV with a 3^rd^ generation assay using CMIA (enzymatic immunoassay with chemiluminescent detection) on the Architect i2000 analyser, by Abbott, USA [28]. The presence or absence of anti-HCV antibodies in the sample was determined based on comparing the value of the chemiluminescent signal in the reaction (RLU) with the cut-off value (CO). A sample was considered positive (reactive) when the sample had a chemiluminescent signal greater than or equal to the cut-off value based on the S/CO (sample/cut-off) formula. Samples with an S/CO < 1 were labelled negative (non-reactive), samples with S/CO values from 1–2 were defined as borderline reactive, and samples with S/CO > 2 were considered positive (reactive). All borderline reactive samples were tested using the confirmation immunoblot test INNO-LIA HCV Score (Fujirebio Europe N.V., Belgium) in the National Reference Laboratory. The test variant requiring a 3-hour incubation of the tested serum was performed.

### Determining HCV RNA

All borderline and positive samples were tested using RT-PCR for direct detection of hepatitis C virus. Isolation of HCV RNA from serum or plasma was performed using the QIAamp® Viral RNA Mini Kit (250) by Qiagen, and amplification with detection on the RotorGene 6000 5plex apparatus for real-time PCR was conducted with the commercial kit artus HCV RG RT-PCR, by QIAGEN.

### HCV genotyping

HCV genotypes were determined in HCV RNA-positive samples. A modification of conventional PCR was used for genotyping, specifically multiplex nested PCR using universal (outer primers) and specific primers (inner primers) positioned in the region of the HCV genome core, with visualization on agarose gel dyed with ethidium bromide (29). The length of the product on the gel corresponded to individual genotypes: 1a-49 bp; 1b-144 bp; 2a-174 bp; 2b-123 bp a 3a-88 bp.

### Statistical analysis

To present the results, the absolute and relative frequencies were used. To determine the differences between groups, the χ2 test of independence on contingency tables and Fisher’s exact test of difference of two relative values were used. The Wilson Score confidence interval method was used for calculating confidence intervals.

The age-adjusted prevalence was calculating using the direct method of adjustment based on the age-specific rates for the sample and age-specific structure of population in the Czech Republic according to the Czech Statistical Office.

The study protocol was approved by Ethics Committee of University Hospital Hradec Kralove, Czech Republic (201502S10R) and investigation was conducted in accordance with the Declaration of Helsinky. Written informed consent was obtained from all participants before study entry.

## Results

### Population characteristics

During a period of 6.5 months, a total of 3,000 adult subjects were enrolled in the study at the three research centres. The firs centre enrolled a total of 2,000 subjects, while the second and third enrolled 500 subjects each. The mean age of the participants was 47.1 years (SD 17.1), ranging from 18 to 90 years (Table 1). The median age was 46 years. The gender and age distribution in the sample corresponded with the distribution of the general adult population. The study included 1,536 (51.2%) females and 1,464 (48.8%) males. In total, 47 injecting drug users were enrolled (1.6%), and this proportion corresponded to the estimated number of injecting drugs users in the Czech adult population. The highest number of injecting drugs users occurred in the 18- to 29-year-old age group. The sample included 1,038 (34.6%) persons with risk behaviours and 1,962 (65.4%) persons without risk factors. Tattooing or piercing was noted in 525 (17.5%) of the enrolled participants. The risk factors for HCV transmission were collected from questionnaires completed by the subjects.

**Table 1:** Demographic characteristics of subjects.

Age category (years)	Number	Male N (%)	Female N (%)	Mean age (± SD)	Drugs users N (%)
18–29	554	273 (49.3)	281 (50.7)	23.2 ± 3.4	20 (3.6)
30–44	866	421 (49.0)	445 (51.0)	37.1 ± 4.2	23 (2.7)
45–59	702	334 (47.6)	368 (52.4)	52.0 ± 4.5	3 (0.4)
≥ 60	878	437 (49.8)	441 (50.2)	68.2 ± 6.1	1 (0.1)
Total	3000	1465 (48.8)	1535 (51.2)	47.1 ± 17.1	47 (1.6)

### Prevalence of anti-HCV-positive sera

The prevalence of anti-HCV-positive persons in the study population was 1.67% (n = 50). The seroprevalence was higher in males than in females (2.39% vs 0.98%; p = 0.0026). The highest prevalence was found in the 30- to 44-year-old age group (3.58%; n = 31). The overall crude prevalence rate was 16.52 per 1,000 and age-adjusted prevalence rate was 16.67 per 1,000. The lowest prevalence was observed in people aged 60 years and older (0.23%; n = 2) (Table 2). Only 2 persons (4%) were aware of their illness prior to the testing. The majority of persons testing positive for anamnestic antibodies had never been diagnosed prior to the testing. When comparing the prevalence by risk factors or behaviour, a higher prevalence was found in persons with risk behaviour (3.46% vs 0.71%). None of the persons working in healthcare (195 people in the entire sample) had positive anti-HCV antibodies. The prevalence in injecting drug users was 51.1% (Table 3). After excluding injecting drug users (n = 47), the prevalence of persons with positive antibodies was 0.88%.

**Table 2:** Prevalence of anti-HCV- positive and HCV RNA-positive subjects by sex/age.

Characteristic	Number (N)	Anti-HCV-positive N (%)	95% CI*	HCV RNA-positive N (%)	95% CI*
**Sex**					
Female	1535	15 (0.98%)	0.59–1.61	6 (0.39%)	0.18–0.85
Male	1465	35 (2.39%)	1.72–3.30	22 (1.50%)	0.99–2.26
**Age (years)**					
18–29	554	11 (1.99%)	1.11–3.52	7 (1.26%)	0.61–2.58
30–44	866	31 (3.58%)	2.53–5.04	18 (2.08%)	1.32–3.26
45–59	702	6 (0.85%)	0.39–1.85	3 (0.43%)	0.15–1.25
60+	878	2 (0.23%)	0.06–0.83	0 (0.0%)	0.0–0.44
Total	3000	50 (1.67%)	1.27–2.19	28 (0.93%)	0.65–1.35

* Confidence Interval.

**Table 3:** Prevalence of anti-HCV- positive and HCV RNA-positive subjects by risk.

Characteristic	Number (N)	Anti-HCV-positive N (%)	95% CI*	HCV RNA-positive N (%)	95% CI*
Risk group	1039	36 (3.46%)	2.51–4.76	22 (2.12%)	1.40–3.19
No risk group	1961	14 (0.71%)	0.43–1.19	6 (0.31%)	0.14–0.67
					
Intravenous drug users	47	24 (51.06%)	37.24–64.72	11 (23.4%)	13.60–37.22

* Confidence Interval.

### Prevalence of HCV–RNA-positive sera

The total prevalence of chronic hepatitis in adults (HCV RNA positivity) in the study population was 0.93%; n = 28. A higher prevalence was found in males compared to females (1.5% vs 0.39%; p = 0.00186). The highest prevalence was found in those aged 30–44 years (2.08%). The lowest prevalence was found in persons aged 45–59 years (0.43%; n = 3). Not a single case of HCV RNA positivity (Table 2) was found in persons aged 60 years and older. The overall crude prevalence rate was 9.33 per 1,000 and age-adjusted prevalence rate was 9.21 per 1,000. None of the HCV RNA-positive persons were aware of their disease prior to their enrolment in the study, and they had no subjective symptoms. When comparing the prevalence by risk factors or behaviour, the highest prevalence was observed in people with high-risk behaviour (2.12% vs 0.31%). There were 20 subjects HCV-RNA positive in the subgroup of persons with history of tattooing or piercing (525 subjects) in our study (3.81% of all subjects in this subgroup of people). The prevalence in injecting drug users was 23.4% (Table 3). After excluding injecting drug users (n = 47) from the analysis, 0.58% of the remaining study population had HCV RNA-positive sera.

### HCV genotyping

All samples that tested positive in the HCV RNA (n = 28) test were genotyped. The virus genotype was determined in 96.4% of the samples; in one sample (3.6%), the genotype was undetermined. The 3a genotype was the most prevalent (46.4%). The other most frequent genotypes included 1a (25.0%) and 1b (25.0%).

## Discussion

The study population of adult persons was a sample of the general Czech population. The study enrolled volunteers with a gender and age distribution corresponding to that of the general adult population. The study population included people exhibiting high-risk behaviours including injecting drug users. There were a total of 47 injecting drug users (1.6%) in the study. No census data are available for the number of injecting drugs users in general population and their proportion is only estimated from 0.4% to 3% [30]. It is a question if number of IDUs in the study is consistent with the general Czech population. The study confirmed a very high risk of hepatitis C infection in injecting drug users. In our study population, the anti-HCV antibody prevalence in injecting drug users was 51.1%. More than half of the injecting drug users had been exposed to hepatitis C infection, and 23% of them had chronic hepatitis C infection.

Also 195 healthcare workers (HCWs) were enrolled in the study. No a single case of hepatitis C infection among them may indicate minimal risk of HCV transmission from patients to medical personnel. The study population included a total of 17.5% of persons who had a history of tattooing or piercing. But there is no evidence of association with HCV infection.

The seroprevalence of anamnestic antibodies against HCV and the prevalence of HCV RNA-positive sera based on age corresponded to findings from European data [8] and were higher in males compared to females (2.4 times and 3.8 times higher, respectively). The highest seroprevalence of hepatitis C was found in persons who were 7–21 years of age in 1992, which is when the testing of blood and organ donors for hepatitis C was introduced (corresponding to the 30- to 44-year-old age group in our study). Therefore, they were not expected to have been infected by contaminated blood products from an HCV-positive donor.

In comparison with data from 2001 (0.2%), over the last 14 years, there has been an increase in the prevalence of anti-HCV anamnestic antibodies in the Czech Republic (1.67%) and in the prevalence of chronic HCV infection in the adult population (0.97%). The highest increase in prevalence was found in the age group of those up to 45 years, with a maximum in the 30- to 44-year-old age group (the 1971–1985 birth cohorts). This finding conflicts with research in the US, which identified baby boomers as the major risk group (the 1945–1965 birth cohorts).

In contrast to the reported [29,30] percentage of patients with HCV infection advancing to the chronic stage, the rate of transition to chronic infection in our study population was only 56%.

We found a higher proportion of the HCV genotype 3a in patients with chronic hepatitis C than in previous years. This result is in contrast with findings from previous years, when GT 1 was clearly predominant (66%) and GT 3 occurred in 31.1% [19,20]. In our study population, the GT3 proportion increased (46.4%), while GT1 decreased (50.0%). The increasing prevalence of GT3 carries a higher risk of more rapid progression of chronic HCV infection, and GT3 can become difficult to treat, especially in cirrhotic patients. Cirrhotic cases associated with GT 3 infection are becoming more prevalent in the Czech Republic. We can expect an increase in the number of patients with liver cirrhosis who need a liver transplant or who develop hepatocellular cancer in the Czech Republic. The burden of chronic hepatitis C may thus be greater than expected and greater than the reported incidence data would warrant.

An integral part of reducing the estimated health burden of chronic hepatitis C is providing more effective screening and increasing the efficacy of chronic infection treatment. The significance of screening has increased with the advent of directly acting antiviral treatment, which has the potential to cure and completely eradicate hepatitis C. Its efficacy is fully dependent on the early identification of persons infected with hepatitis C [25,31].

The limitation of the study is the risk for selection bias as the population sample was recruited at a research center after using advertising campaign in the media. The study could be biased by the higher proportion of health care workers in the study population, 195 out of 3,000 subjects. However, none of them were anti-HCV positive. Furthermore, the enrolment of 47 injecting drug users could have influenced the results, accounting for the fact that there are drug users in the general population.

## Conclusions

Compared to the last available official data from over 10 years ago, there has been an increase in the seroprevalence of hepatitis C in the adult population. This finding corresponds to the increased incidence of hepatitis C, when in 2015, there was an increase to 9.1/100,000 inhabitants (7.7/100,000 in 2011). A higher prevalence of hepatitis C has been confirmed in injecting drug users, persons with risk behaviour, and males. Additionally, a transition to chronic disease was documented in 56% of patients with anamnestic antibodies, confirming their exposure to hepatitis C. The highest prevalence of chronic hepatitis C was found in adults aged 30–44 years. In the last 10 years, there has been an increase in the incidence of GT 3a of more than 10%. With a slight delay, this change in genotype distribution follows the trend in Western Europe and North America. A chronic hepatitis C infection pandemic can also be expected in the Czech Republic.

Based on our results, we anticipate that in the Czech population, there are more than 140,000 persons who have been infected with hepatitis C, more than 80,000 persons who live with chronic hepatitis C, and more than 50,000 of those persons who are in the 30- to 44-year-old age group. These results are higher than the reported incidence, and this difference could be caused by underreporting. The introduction of screening for selected age groups, early diagnosis and an increase in the awareness of the infection within this population could improve the current status of hepatitis C control. The screening program needs to be individualized for specific population based on risk factors and age. The identification of the individual factors is crucial to make appropriate screening interventions. The results of our study suggest the feasibility of not only extending screening strategies to persons with risk behaviour but also introducing birth cohort HCV screening for the Czech adult population.
